# Neutralizing Activity Against SARS-CoV-2 Delta and Omicron Variants Following a Third BNT162b2 Booster Dose According to Three Homologous or Heterologous COVID-19 Vaccination Schedules

**DOI:** 10.3389/fcimb.2022.948014

**Published:** 2022-07-11

**Authors:** Ju-yeon Choi, Young Jae Lee, Jae-Hoon Ko, Su-Hwan Kim, Hye-Jin Kim, Hye Won Lee, Hyeonji Jeong, Tae-Yong Kim, Yeong Gyeong Jang, Hyo-jeong Hong, Min-Seong Kim, Sang Eun Lee, Yong Guan Kim, Eun Joo Chung, Heeji Lim, Sundong Jang, Kwangwook Kim, Sung Soon Kim, Jin Young Ahn, Jun Yong Choi, Yong Chan Kim, Yoon Soo Park, Kyong Ran Peck, Byoungguk Kim

**Affiliations:** ^1^ Center for Vaccine Research, National Institute of Infectious Diseases, Korea National Institute of Health, Korea Disease Control and Prevention Agency, Cheongju, South Korea; ^2^ Division of Infectious Diseases, Department of Medicine, Samsung Medical Center, Sungkyunkwan University School of Medicine, Seoul, South Korea; ^3^ Department of Internal Medicine, Severance Hospital, Yonsei University College of Medicine, Seoul, South Korea; ^4^ Division of Infectious Disease, Department of Internal Medicine, Yongin Severance Hospital, Yonsei University College of Medicine, Yongin, South Korea

**Keywords:** neutralizing activity, Omicron variant, Delta variant, SARS-CoV-2, vaccine

## Abstract

With the emergence and rapid spread of severe acute respiratory syndrome coronavirus 2 (SARS-CoV-2) Delta and Omicron variants, escaping vaccine-induced immunity is a concern. Three vaccination schedules, homologous or heterologous, have been initially applied due to an insufficient supply of vaccines in Korea. We investigated neutralizing activities against Omicron and Delta variants in each schedule. Three schedules using three doses of the BNT162b2 (BNT) or the ChAdOx1 (ChAd) vaccines include ChAd-ChAd-BNT, ChAd-BNT-BNT, and BNT-BNT-BNT. Neutralizing activities were evaluated using plaque-reduction neutralization test (PRNT) against wild type (WT) SARS-CoV-2, Delta variant, and Omicron variant. A total of 170 sera from 75 participants were tested, and the baseline characteristics of participants were not significantly different between groups. After the 2nd vaccine dose, geometric mean titers of PRNT ND_50_ against WT, Delta, and Omicron were highest after ChAd-BNT vaccination (2,463, 1,097, and 107) followed by BNT-BNT (2,364, 674, and 38) and ChAd-ChAd (449, 163, and 25). After the 3rd dose of BNT, the increase of PRNT ND_50_ against WT, Delta, and Omicron was most robust in ChAd-ChAd-BNT (4,632, 988, and 260), while the BNT-BNT-BNT group showed the most augmented neutralizing activity against Delta and Omicron variants (2,315 and 628). ChAd-BNT-BNT showed a slight increase of PRNT ND_50_ against WT, Delta, and Omicron (2,757, 1,279, and 230) compared to the 2nd dose. The results suggest that a 3rd BNT booster dose induced strengthened neutralizing activity against Delta and Omicron variants. The waning of cross-reactive neutralizing antibodies after the 3rd dose and the need for additional boosting should be further investigated.

## Introduction

Approximately 2 years after the COVID-19 pandemic began, the rapid spread of the Omicron (B.1.1.529) variant, a newly reported variant of concern (VOC), became a high-priority issue worldwide (Gu et al., 2022; WHO, 2022). Omicron was first reported in South Africa on November 24, 2021 (WHO, 2022), and the first Korean case was identified on November 25, 2021 (Lee et al., 2021). Within 2 months of its introduction, the Omicron variant became the dominant global strain (Lee et al., 2021; Song et al., 2022). Similar to the Delta (B.1.617.2) variant that sparked the global COVID-19 outbreak in late 2021, Omicron escapes vaccine-induced immunity further *via* multiple mutations in key epitopes for neutralizing antibodies (Andrews et al., 2022; Buchan et al., 2022; Gruell et al., 2022). In the Republic of Korea, nationwide COVID-19 vaccination using the BNT162b2 (BNT; Comirnaty, Pfizer/BioNTech, Mainz, Germany) or the ChAdOx1 (ChAd; Vaxzevria, to AstraZeneca, Oxford, UK) vaccines was implemented in early 2021 with three major homologous or heterologous two-dose vaccination schedules, including ChAd-ChAd, ChAd-BNT, and BNT-BNT, according to the vaccine-induced adverse events (especially severe reactogenicity and vaccine-induced immune thrombotic thrombocytopenia after ChAd) and vaccine supply (Bae et al., 2021; Kim et al., 2021; Yang et al., 2021; Bae et al., 2022). From late 2021, individuals were encouraged to receive a 3rd dose of BNT to overcome the immune escape of Delta and Omicron. In this study, we investigated and compared neutralizing activities against Omicron and Delta variants after three-dose vaccination schedules.

## Material and methods

### The Vaccination Group for Analysis

For the evaluation of neutralizing activities against the Omicron and Delta variants, we selected 170 sera from 75 participants of a nationwide multicenter prospective cohort (50 samples from 35 ChAd-ChAd-BNT participants, 60 samples from 20 ChAd-BNT-BNT participants, and 60 samples from 20 BNT-BNT-BNT participants) (Bae et al., 2022). Details are presented in **Supplementary Table S1**. All participants provided written informed consent, and the study protocol was approved by the institutional review board of each participating hospital. The laboratory procedures for the plaque reduction neutralizing test (PRNT) and methods for statistical analysis are presented in the **Supplementary Material**.

### Plaque Reduction Neutralization Test

To evaluate the functionality of the vaccine-induced antibody response, we performed PRNT against wild type (WT), Delta variant, and Omicron variant of severe acute respiratory syndrome virus 2 (SARS-CoV-2) (Harcourt et al., 2020; Okba et al., 2020). SARS-CoV-2 dilutions to 40~50 PFU/well (WT, βCoV/Korea/KCDC03/2020 NCCP No. 43326; Omicron, Gyeonggi GRA: B.1.1.529 NCCP No. 43408; Delta, Gyeonggi GK: AY.69 NCCP No. 43409) were prepared. Vero E6 cells were inoculated with serum and virus mixtures on a 12-well plate and incubated at 37°C, 5% CO_2_ for 1 h. After inoculums were removed, cells were overlaid with 1 ml of modified Eagle’s medium (Gibco, Gaithersburg, MD, USA) containing 0.75% agarose and 2% fetal bovine serum (Gibco). The plates were incubated at 37°C with 5% CO_2_ for 2 or 3 days. Stain solution (0.07% crystal violet, 10% formaldehyde, and 5% ethanol) was then added to the cells, and the visualized plaques were counted. The 50% neutralizing dose (ND_50_) titer was calculated using the Karber formula.

### Statistical Analysis

For the three-group comparison of baseline characteristics and clinical variables, one-way analysis of variance (ANOVA) was used for continuous variables, and the Chi-square test was used for categorical variables. For the two-group comparison of PRNT ND_50_ results, Student’s *t*-test was used. A linear regression model was used to evaluate the correlations between PRNT titers against WT and VOCs. For the interpretation of the correlation coefficient, *R* ^2^ ≥ 0.7 was considered a strong correlation, *R* ^2^ ≥ 0.4 a moderate correlation, and *R* ^2^ < 0.47 a week correlation (Schober et al., 2018). All *p*-values were two tailed, and values <0.05 were considered to be statistically significant. GraphPad Prism version 8.0 (GraphPad Software, San Diego, CA, USA) was used for the analysis and graph plotting of the results.

## Results

**Figure 1:**
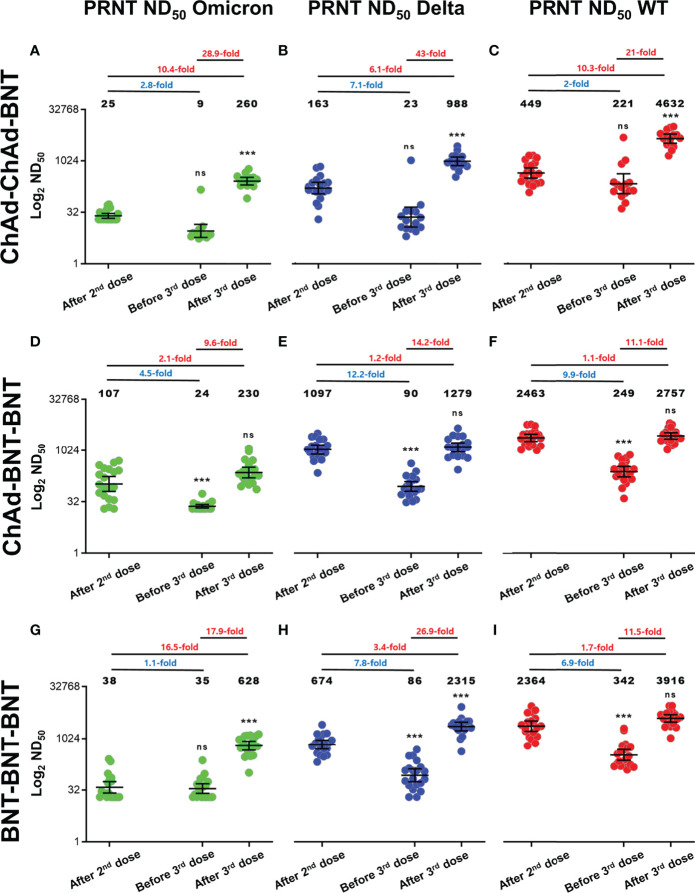
PRNT ND_50_ against Delta and Omicron variants compared to Wild-type SARS-CoV-2 for each vaccination schedule. **(A–I)** Log_2_ PRNT ND_50_ for Omicron, Delta, and WT SARS-CoV-2 is presented for each vaccination group including ChAd-ChAd-BNT **(A–C)**, ChAd-BNT-BNT **(D–F)**, and BNT-BNT-BNT **(G–I)**. All values are expressed as the geometric mean titer (GMT) of each group, and the error bar indicates the 95% confidence interval (CI). Fold notation according to color expresses decrease and increase. Blue text indicates fold reduction; red text indicates fold increase. Results of statistical significance compared with the sera after the 2nd dose are presented above sera before the 3rd dose and after the 3rd dose (asterisks (^***^) denotes statistically significant difference; ns denotes not significant). Green dots reflect Omicron variants, blue dots reflect Delta variants, and red dots reflect WT SARS-CoV-2 (GraphPad Software, San Diego, CA, USA). PRNT, plaque reduction neutralizing test; ND_50_, 50% neutralization dose; WT, wild type; ChAd, ChAdOx1 vaccine; BNT, BNT162b2 vaccine.

Baseline characteristics of participants are presented in **Supplementary Table S1**, and they were not significantly different between groups. At 2–3 weeks after the 2nd vaccine dose, the geometric mean titer (GMT) of PRNT ND_50_ against WT, Delta, and Omicron was highest after the ChAd-BNT vaccination schedule (2,463, 1,097, and 107) followed by the BNT-BNT schedule (2,364, 674, and 38) and the ChAd-ChAd schedule (449, 163, and 25) (**Figure 1**). Approximately 5 months after the 2nd dose, PRNT ND_50_ waned, especially with the ChAd-BNT schedule. However, the absolute values of GMT were lowest in the ChAd-ChAd group.

After the 3rd dose of BNT, increases in PRNT ND_50_ against WT, Delta, and Omicron differed among vaccination groups. In the ChAd-ChAd-BNT group, robust increases of PRNT ND_50_ against WT (10.3-fold), Delta (6.1-fold), and Omicron (10.4-fold) were observed, compared to peaks after the 2nd dose (all *p* < 0.05). In the ChAd-BNT-BNT group, increases of PRNT ND_50_ against WT (1.1-fold), Delta (1.2-fold), and Omicron (2.1-fold) were not statistically significant compared to those after the 2nd dose (all *p* > 0.05). In the BNT-BNT-BNT group, the increases in PRNT ND_50_ against Delta (3.4-fold) and Omicron (16.5-fold) were significant, but the PRNT ND_50_ against WT (1.7-fold) was not.

After the 2nd vaccine dose, PRNT ND_50_ in the ChAd-BNT-BNT group against WT, Delta, and Omicron was higher than in other groups such as ChAd-ChAd-BNT and BNT-BNT-BNT. Since then, the BNT-BNT-BNT group showed overall robust neutralizing activities except for PRNT ND_50_ in the ChAd-BNT-BNT group against Delta (GMT 90, before 3rd dose) and in the ChAd-ChAd-BNT group against WT (GMT 4632, after 3rd dose) (**Supplementary Figure S1**).

**Figure 2:**
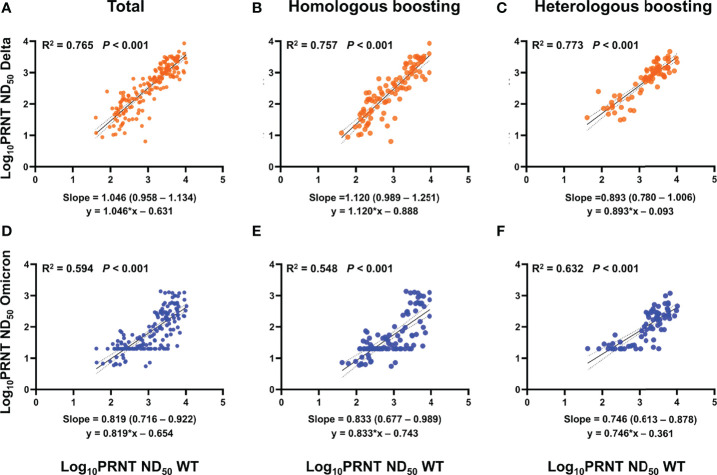
PRNT ND_50_ correlation between WT and Delta or Omicron variant. **(A–F)** Log_10_ PRNT ND_50_ among strains was compared using a linear regression model. When interpreting the correlation coefficient, *R* ^2^ ≥ 0.7 was considered a strong correlation, *R* ^2^ ≥ 0.4 a moderate correlation, and *R* ^2^ < 0.47 a week correlation (Schober et al., 2018). **(A–C)** WT and Delta variants show a consistently strong correlation regardless of vaccination groups. **(D–F)** Linear correlation between PRNT ND_50_ against WT and Omicron was also noticed, but the coefficient of determination was lower than that between WT and Delta (moderate correlation). Homologous boosting included sera after BNT-BNT, BNT-BNT-BNT, and ChAd-ChAd, while heterologous boosting included sera after ChAd-BNT, ChAd-BNT-BNT, and ChAd-ChAd-BNT. PRNT, plaque reduction neutralizing test; ND_50_, 50% neutralization dose; WT, wild type; ChAd, ChAdOx1 vaccine; BNT, BNT162b2 vaccine.

To evaluate whether neutralizing activities against WT reflect those against Delta or Omicron, PRNT ND_50_ results against strains were compared using a log scale (**Figure 2**; **Supplementary Figures S2, S3**). Among vaccination groups, PRNT ND_50_ against WT consistently correlated well with PRNT ND_50_ against Delta (*R* ^2^ = 0.765, *p* < 0.001) (Schober et al., 2018). A linear correlation between PRNT ND_50_ against WT and Omicron was also observed, but the coefficient of determination was lower than that between WT and Delta (*R* ^2^ = 0.594, *p* < 0.001) and different between vaccination groups. The *R* ^2^ between PRNT ND_50_ against WT and Omicron was lowest in the BNT-BNT-BNT group (0.414), followed by the ChAd-BNT-BNT (0.612) and the ChAd-ChAd-BNT (0.768) groups.

## Discussion

Several escapes from vaccine-induced immunity are a major problem for emerging VOCs, especially for the Omicron variant. Although more than 80% of the South Korean population received at least two doses of COVID-19 vaccines, the Omicron outbreak surged in March 2022. Previous studies suggest that a 3rd dose of mRNA vaccine provides higher protection against Omicron variants (Andrews et al., 2022; Garcia-Beltran et al., 2022), but the effect of vaccination based on schedule needs to be further investigated.

Of note, an increase in neutralizing activity after the 3rd dose against Delta and Omicron was observed in all three vaccination groups, but the magnitude of increase was different between groups. After a 3rd BNT dose, the homologous BNT-BNT-BNT group offers the most robust increase of neutralizing activity against Delta (3.4-fold) and Omicron (16.5-fold), in contrast to a modest increase against WT (1.7-fold). A 3rd BNT dose induced an overall robust antibody response in the ChAd-ChAd-BNT group, but the increase of neutralizing activity against Delta (6.1-fold) and Omicron (10.4-fold) was not higher than that against WT (10.3-fold). In the ChAd-BNT-BNT group, the increase in neutralizing activity against WT (1.1-fold) and Delta (1.2-fold) was not significant compared to the 2nd dose, while the PRNT ND_50_ against Omicron showed a 2.1-fold increase. Our PRNT data, which show that the BNT-BNT-BNT group has a higher neutralizing activity than the ChAd-ChAd-BNT group, support a vaccine effectiveness study conducted in the United Kingdom, which found that the BNT-BNT-BNT group (67.2%; 95% CI, 66.5–67.8) had higher vaccine effectiveness against the Omicron variant than the ChAd-ChAd-BNT group (62.4%; 95% CI, 61.8–63.0) (Andrews et al., 2022). Maturation of neutralizing antibodies after a 3rd dose of homologous mRNA vaccination was previously published (Garcia-Beltran et al., 2022), but the effect of heterologous boosting has not been fully investigated. In the present analysis, we noticed that cross-reactive neutralizing activity against Delta and Omicron variants was most effectively induced by homologous boosting with three doses of mRNA vaccines. Although heterologous 2nd dose of BNT following ChAd (ChAd-BNT) induced higher cross-reactivity than homologous immunizations (BNT-BNT or ChAd-ChAd), antibody titers decreased most rapidly. The 3rd dose of BNT also did not induce a robust boosting response compared to the 2nd dose. In addition to the longevity of cross-reactive neutralizing antibodies after the 3rd dose, further maturation of neutralizing antibodies might occur after a 4th dose of vaccine, which needs to be further investigated in each vaccination group.

In addition, we also demonstrated that the correlation between neutralizing activity against WT and Omicron was less linear than that between WT and Delta. A previous study using pseudo-virus also suggested a poor correlation between WT and Omicron PRNT (Garcia-Beltran et al., 2022). These findings suggest that measuring neutralizing antibodies against WT is not sufficient to predict protection against Omicron and future-emerging VOCs. Also, binding antibody test kits developed based on the WT SARS-CoV-2 also need to be validated. This is especially important considering the outbreak situation following the initial wave caused by Omicron. Subvariants of Omicron such as BA2.12.1, BA.4, and BA.5 quickly have been surging to dominance in many parts of the world and might be better at evading vaccine-induced immunity.

The present study has several limitations and strengths. First, the number of participants was limited as PRNT requires time and skilled personnel. However, most recently, studies evaluating neutralizing activities utilized pseudovirus neutralizing assays or surrogate virus-neutralizing tests (Garcia-Beltran et al., 2022). Our present analyses using the PRNT method help reflect the actual protective immunity against Delta and Omicron variants. Second, as all participants were relatively young and healthy, our data might not reflect those with high comorbidities or those of older age. Nevertheless, a homogenous study population would be suitable for comparing the vaccine-induced immunity of each vaccination group.

In conclusion, a 3rd BNT booster dose induced strengthened neutralizing activity against Delta and Omicron variants. The waning of cross-reactive neutralizing antibodies after the 3rd dose and the need for additional boosting should be further investigated.

## Data Availability Statement

The original contributions presented in the study are included in the article/**Supplementary Material**. Further inquiries can be directed to the corresponding authors.

## Ethics Statement

Institutional review board of Samsung Medical Center, Yongin Severance Hospital, and Sinchon Severance Hospital approved this study.

- Division of Infectious Diseases, Department of Medicine, Samsung Medical Center, Sungkyunkwan University School of Medicine, Seoul, Republic of Korea.

- Department of Internal Medicine, Severance Hospital, Yonsei University College of Medicine, Seoul, Republic of Korea.

- Division of Infectious Disease, Department of Internal Medicine, Yongin Severance Hospital, Yonsei University College of Medicine, Yongin, Republic of Korea. The patients/participants provided their written informed consent to participate in this study.

## Author Contributions

JC, YL, J-HK, KP, BK, and SK were involved in the design of this study. J-HK, JA, JC, YCK, YP, and KP enrolled participants and collected specimens. S-HK, HJ, T-YK, M-SK, SL, YGJ, HH, YGK, EJC, HJL, SJ, and KK performed the experiments. H-JK, HWL, and J-HK assembled the data. JC, YL, J-HK, KP, and BK were involved in writing. All authors crucially approved and revised the manuscript.

## Funding

This study was supported by an intramural fund (No. 4800-4861-313) from the Korea National Institute of Health, research program funds (#2021-ER2601-00 and 2021-ER2303-00) by the Korea Disease Control and Prevention Agency, and a Samsung Medical Center Grant (#SMO1220371).

## Conflict of Interest

The authors declare that the research was conducted in the absence of any commercial or financial relationships that could be construed as a potential conflict of interest.

## Publisher’s Note

All claims expressed in this article are solely those of the authors and do not necessarily represent those of their affiliated organizations, or those of the publisher, the editors and the reviewers. Any product that may be evaluated in this article, or claim that may be made by its manufacturer, is not guaranteed or endorsed by the publisher.

## References

[B1] Andrews N. Stowe J. Kirsebom F. Toffa S. Rickeard T. Gallagher E.. (2022). Covid-19 Vaccine Effectiveness Against the Omicron (B.1.1.529) Variant. N Engl. J. Med. 386 (16), 1532–1546. doi: 10.1056/NEJMoa2119451 35249272 PMC8908811

[B2] Bae S. Ko J. H. Choi J. Y. Park W. J. Lim S. Y. Ahn J. Y.. (2022). Heterologous ChAdOx1 and BNT162b2 Vaccination Induces Strong Neutralizing Antibody Responses Against SARS-CoV-2 Including Delta Variant With Tolerable Reactogenicity. Clin. Microbiol. Infect. doi: 10.1016/j.cmi.2022.04.019 PMC9117169 35598855

[B3] Bae S. Lee Y. W. Lim S. Y. Lee J. H. Lim J. S. Lee S.. (2021). Adverse Reactions Following the First Dose of ChAdOx1 Ncov-19 Vaccine and BNT162b2 Vaccine for Healthcare Workers in South Korea. J. Korean Med. Sci. 36 (17), e115. doi: 10.3346/jkms.2021.36.e115 33942579 PMC8093607

[B4] Buchan S. A. Chung H. Brown K. A. Austin P. C. Fell D. B. Gubbay J. B.. (2022). Effectiveness of COVID-19 Vaccines Against Omicron or Delta Symptomatic Infection and Severe Outcomes. medRxiv 2021. doi: 10.1101/2021.12.30.21268565 PMC9500552 36136332

[B5] Garcia-Beltran W. F. St Denis K. J. Hoelzemer A. Lam E. C. Nitido A. D. Sheehan M. L.. (2022). mRNA-Based COVID-19 Vaccine Boosters Induce Neutralizing Immunity Against SARS-CoV-2 Omicron Variant. Cell 185 (3), 457–466.e454. doi: 10.1016/j.cell.2021.12.033 34995482 PMC8733787

[B6] Gruell H. Vanshylla K. Tober-Lau P. Hillus D. Schommers P. Lehmann C.. (2022). mRNA Booster Immunization Elicits Potent Neutralizing Serum Activity Against the SARS-CoV-2 Omicron Variant. Nat. Med. 28 (3), 477–480. doi: 10.1038/s41591-021-01676-0 35046572 PMC8767537

[B7] Gu H. Krishnan P. Ng D. Y. M. Chang L. D. J. Liu G. Y. Z. Cheng S. S. M.. (2022). Probable Transmission of SARS-CoV-2 Omicron Variant in Quarantine Hotel, Hong Kong, China, November 2021. Emerg. Infect. Dis. 28 (2), 460–462. doi: 10.3201/eid2802.212422 34860154 PMC8798678

[B8] Harcourt J. Tamin A. Lu X. Kamili S. Sakthivel S. Murray J.. (2020). Severe Acute Respiratory Syndrome Coronavirus 2 From Patient With Coronavirus Disease, United States. Emerging Infect. Dis. J. 26 (6):1266–1273. doi: 10.3201/eid2606.200516 PMC7258473 32160149

[B9] Kim G. Choi E. J. Park H. S. Lee J. H. Lee J. H. Lee K. H. (2021). A Case Report of Immune Thrombocytopenia After ChAdOx1 Ncov-19 Vaccination. J. Korean Med. Sci. 36 (43), e306. doi: 10.3346/jkms.2021.36.e306 34751013 PMC8575766

[B10] Lee J. J. Choe Y. J. Jeong H. Kim M. Kim S. Yoo H.. (2021). Importation and Transmission of SARS-CoV-2 B.1.1.529 (Omicron) Variant of Concern in Korea, November 2021. J. Korean Med. Sci. 36 (50), e346. doi: 10.3346/jkms.2021.36.e346 34962117 PMC8728587

[B11] Okba N. M. A. Müller M. Li W. Wang C. GeurtsvanKessel C. Corman V.. (2020). Severe Acute Respiratory Syndrome Coronavirus 2–Specific Antibody Responses in Coronavirus Disease Patients. Emerging Infect. Dis. J. 26 (7), 1478. doi: 10.3201/eid2607.200841 PMC7323511 32267220

[B12] Schober P. Boer C. Schwarte L. A. (2018). Correlation Coefficients: Appropriate Use and Interpretation. Anesth. Analgesia 126 (5), 1763–1768. doi: 10.1213/ane.0000000000002864 29481436

[B13] Song J. S. Lee J. Kim M. Jeong H. S. Kim M. S. Kim S. G.. (2022). Serial Intervals and Household Transmission of SARS-CoV-2 Omicron Variant, South Korea 2021. Emerg. Infect. Dis. 28 (3), 756–759. doi: 10.3201/eid2803.212607 35107418 PMC8888239

[B14] WHO (2022). Tracking SARS-CoV-2 Variants. Available at: http://www.who.int/en/activities/tracking-SARS-CoV-2-variants (Accessed April 22, 2022).

[B15] Yang J. Ko J. H. Baek J. Y. Hong J. Ha S. Lee B.. (2021). Effects of Short-Term Corticosteroid Use on Reactogenicity and Immunogenicity of the First Dose of ChAdOx1 Ncov-19 Vaccine. Front. Immunol. 12. doi: 10.3389/fimmu.2021.744206 PMC8493039 34630425

