# Supplementary material for: Neutralizing Activity Against SARS-CoV-2 Delta and Omicron Variants Following a Third BNT162b2 Booster Dose According to Three Homologous or Heterologous COVID-19 Vaccination Schedules
Source: Front Cell Infect Microbiol. 2022 Jul 11;12:948014. doi: 10.3389/fcimb.2022.948014 (PMC9309354; doi:10.3389/fcimb.2022.948014)
Supplement: Supplementary file 1 [file DataSheet_1.docx]

**Supplementary Table 1. Baseline characteristics of study participants**

| **Characteristics** | **Total**  (n = 75) | **ChAd-ChAd-BNT**  (n = 35) | **ChAd- BNT –BNT**  (n = 20) | **BNT- BNT- BNT**  (n = 20) | ***P* value** |
| --- | --- | --- | --- | --- | --- |
| **Age, years** | 38.7 ± 10.0 | 39.9 ± 9.7 | 39.0 ± 9.8 | 36.3 ± 10.7 | 0.446 |
| **Male sex** | 18 (24.0) | 10 (28.6) | 3 (15.0) | 5 (25.0) | 0.522 |
| **BMI, kg/m^2^** | 22.1 ± 2.6 | 21.9 ± 2.9 | 22.6 ± 2.3 | 22.1 ± 2.4 | 0.661 |
| **Comorbidity, any** | 11 (14.7) | 5 (14.3) | 4 (20.0) | 2 (10.0) | 0.668 |
| Hypertension | 2 (2.7) | 2 (5.7) | 0 (0.0) | 0 (0.0) | 0.309 |
| Thyroid | 2 (2.7) | 0 (0.0) | 1 (5.0) | 1 (5.0) | 0.407 |
| Pulmonary | 1 (1.3) | 1 (2.9) | 0 (0.0) | 0 (0.0) | 0.560 |
| Gastrointestinal | 1 (1.3) | 1 (2.9) | 0 (0.0) | 0 (0.0) | 0.560 |
| Liver | 1 (1.3) | 1 (2.9) | 0 (0.0) | 0 (0.0) | 0.560 |
| Others | 7 (9.3) | 4 (11.4) | 2 (10.0) | 1 (5.0) | 0.728 |
| **Sampling number** |  |  |  |  |  |
| After 2^nd^ dose^†^ | 60 | 20^*^ | 20 | 20 | NA |
| Before 3^rd^ dose^‡^ | 55 | 15^*^ | 20 | 20 | NA |
| After 3^rd^ dose^†^ | 55 | 15^*^ | 20 | 20 | NA |

Data are expressed as the number (%) of participants or mean ± SD. The interval between the 2^nd^ dose and the 3^rd^ dose was less than 6 months, and blood samples were collected at 2-3 weeks and 5 months after the 2^nd^ dose, at 2-3 weeks after the 3^rd^ dose. ^*^Sera for the present analysis were selected by the order of arrival at the laboratory, and participants of the ChAd-ChAd-BNT group were different between first and second/third sampling points. ^†^2-3 weeks after each dose. ^‡^About 5 months after the 2^nd^ dose.

Abbreviations: ChAd, ChAdOx1 vaccine; BNT, BNT162b2 vaccine; BMI, body mass index; NA, not applicable


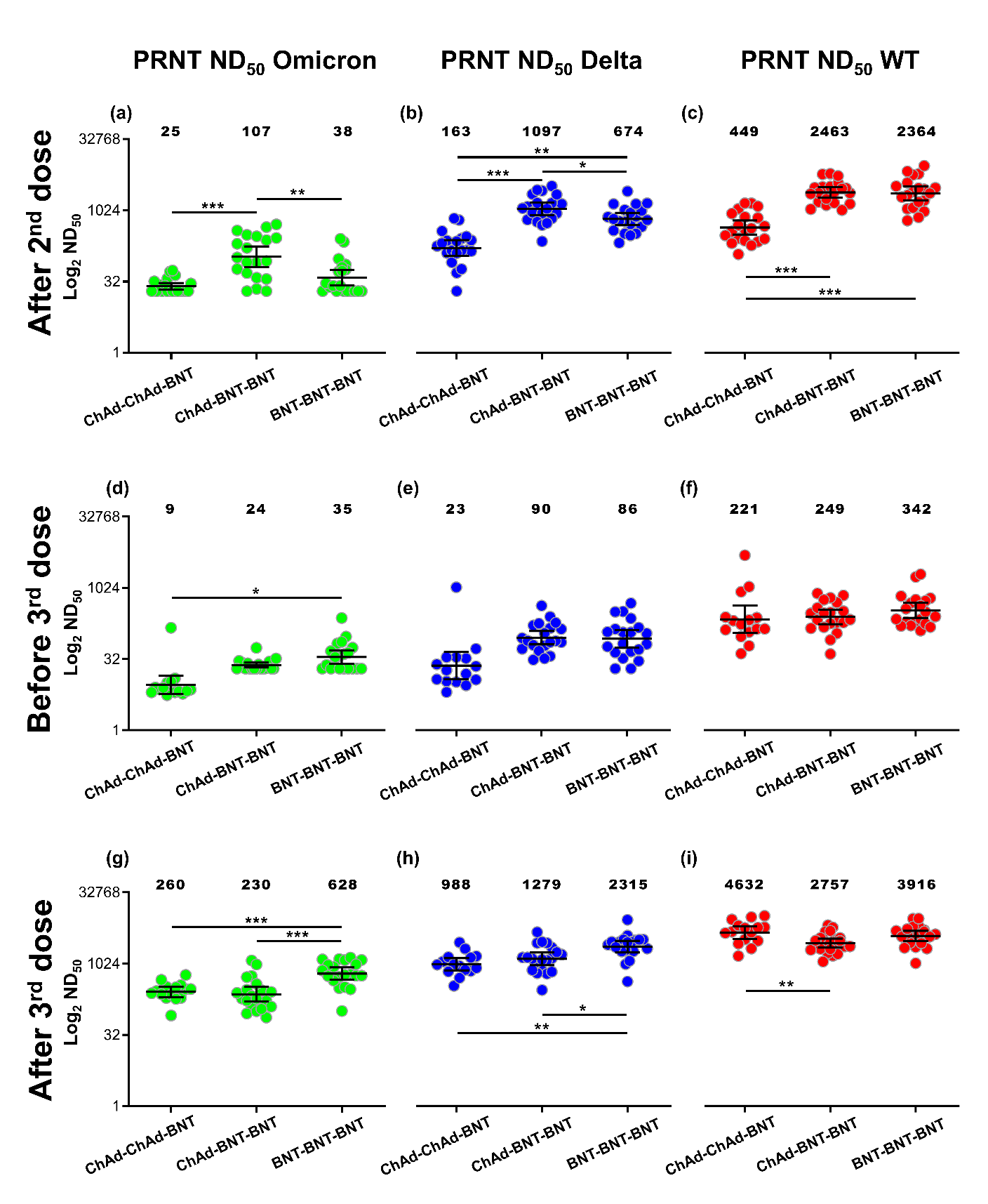


**Supplementary Figure 1. PRNT ND_50_ against Delta and Omicron variants compared to Wild-type SARS-CoV-2 for each time point.**

(a-i) Log_2_ PRNT ND_50_ for Omicron, Delta, and WT SARS-CoV-2 is presented for each time point including after 2^nd^ dose (a-c), before 3^rd^ dose (d-f), and after 3^rd^ dose (g-i). All values are expressed as the geometric mean titer (GMT) of each group, and the error bar indicates the 95% confidence interval (CI). Results of statistical significance compared with the sera of each vaccination schedule with the sera of every other vaccination schedule (*^***^*denotes statistically significant difference). Green dots reflect Omicron variants, blue dots reflect Delta variants, and red dots reflect WT SARS-CoV-2 (GraphPad Software, San Diego, CA, USA).

Abbreviations: PRNT, plaque reduction neutralizing test; ND_50_, 50% neutralization dose; WT, wild-type; ChAd, ChAdOx1 vaccine; BNT, BNT162b2 vaccine

**
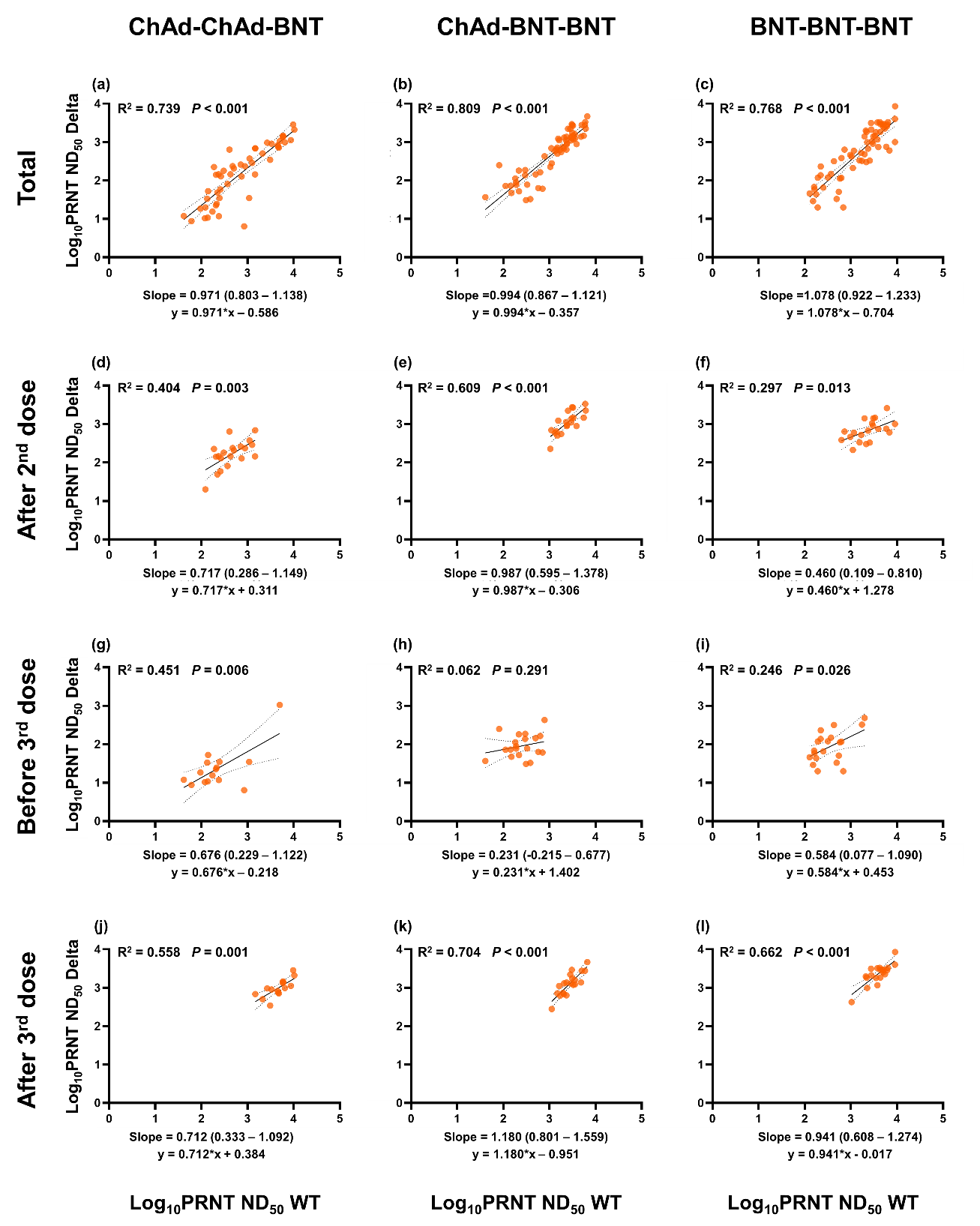
**

**Supplementary Figure 2. PRNT ND_50_ correlation between WT and Delta variant for each point.**

Log_10_ PRNT ND_50_ among strains were compared using a linear regression model. WT and Delta variant show consistently strong correlation at most sampling points.

Abbreviations: PRNT, plaque reduction neutralizing test; ND_50_, 50% neutralization dose; WT, wild-type; ChAd, ChAdOx1 vaccine; BNT, BNT162b2 vaccine


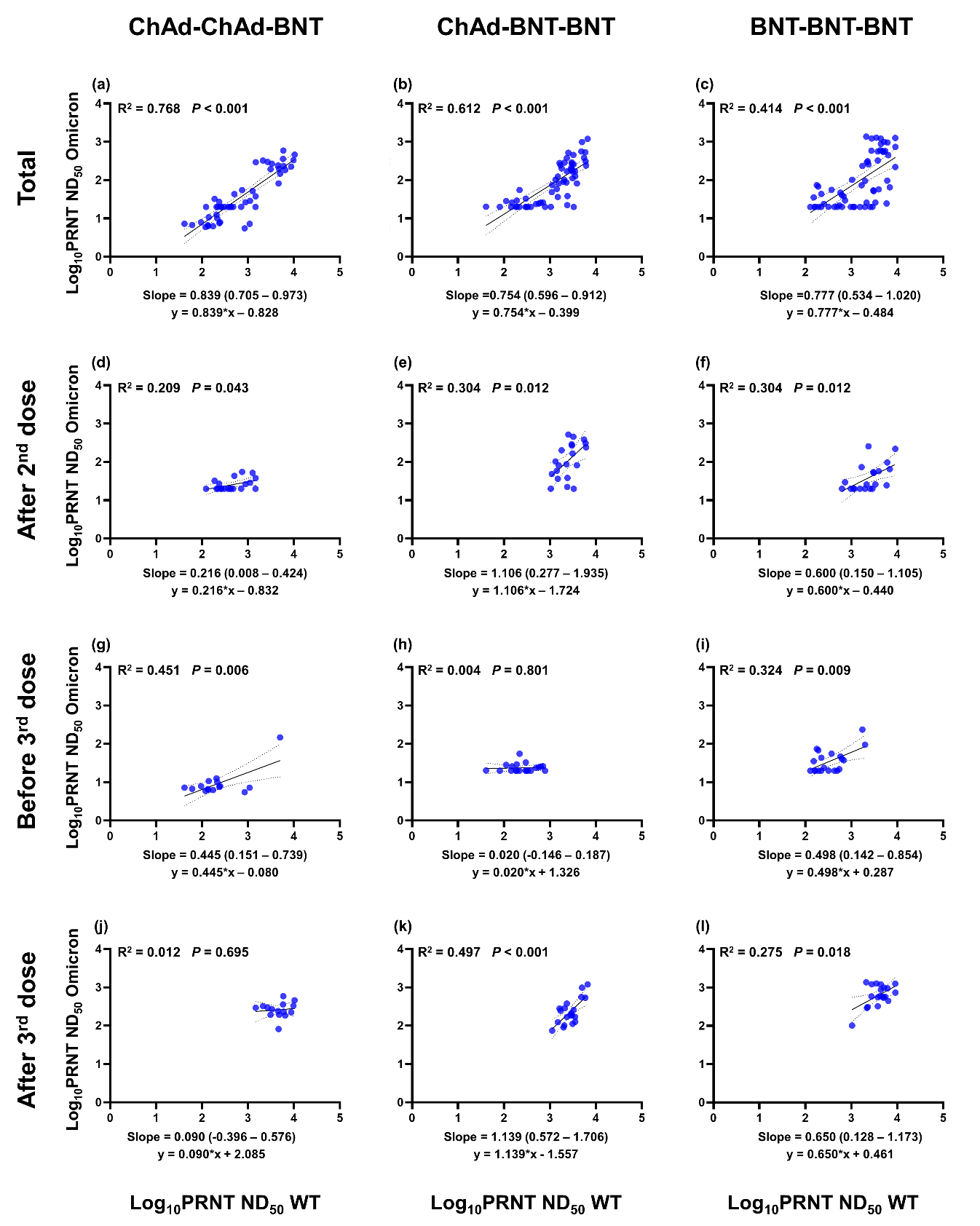


**Supplementary Figure 3. PRNT ND_50_ correlation between WT and Omicron variant for each point.**

Log_10_ PRNT ND_50_ among strains were compared using a linear regression model. WT and Omicron variant show relatively poor correlation, compared to those between WT and Delta variant (moderate correlation).

Abbreviations: PRNT, plaque reduction neutralizing test; ND_50_, 50% neutralization dose; WT, wild-type; ChAd, ChAdOx1 vaccine; BNT, BNT162b2 vaccine
